# How Sugar Labeling Affects Consumer Sugar Reduction: A Case of Sucrose Grade Labels in China

**DOI:** 10.3390/foods13121803

**Published:** 2024-06-08

**Authors:** Yijing Xin, Jiping Sheng, Fujin Yi, Yang Hu

**Affiliations:** 1 School of Agricultural Economics and Rural Development, Renmin University of China, Beijing 100872, China; xinyijing@ruc.edu.cn (Y.X.); shengjiping@126.com (J.S.); 2 School of Public Affairs, Zhejiang University, Hangzhou 310058, China; yifujin@zju.edu.cn; 3 College of Economics and Management Department, Nanjing Agricultural University, Nanjing 210095, China

**Keywords:** sugar labeling, sucrose grade labels, willingness to pay, consistency of sugar control behavior, China

## Abstract

The effectiveness of sugar labeling depends not only on direct sugar reduction but also on the extent to which compensatory eating occurs. This study focuses on the use of sucrose grade labels in the Chinese market to investigate not only consumers’ willingness to pay (WTP) for different sucrose labels but also the consistency of their sugar control behavior when confronted with unlabeled processed foods. The findings reveal that consumers are willing to pay approximately 4%, 7%, and 7% more for yogurt labeled as “low sucrose”, “no sucrose”, and “no sucrose with sugar substitutes”, respectively, compared to yogurt labeled as “regular sucrose.” Furthermore, when subsequently presented with unlabeled toast, a significant proportion of consumers who initially chose “no sucrose” yogurt continued to select wholewheat toast, which contains less sugar than white and coconut toast. This indicates their commitment to maintaining their sugar control behavior. The study provides valuable experimental evidence for researchers, food manufacturers, and policymakers regarding the efficacy of sucrose grade labels. In particular, it offers policymakers insights into guiding consumers to promote sustainable healthy diets.

## 1. Introduction

Sugar labeling has been implemented worldwide as a policy tool to discourage excessive sugar consumption with adverse health consequences [1]. Labels aim to increase consumer’s awareness and understanding of added sugars and to guide them to choose sugar-reduced products [2,3,4]. However, the effectiveness of food labels, such as sugar labeling, depends on the extent to which compensatory eating occurs [5]. If consumers who choose sugar-reduced foods still consume other high-sugar products after labeling interventions, sugar intake will not decrease, and labeling will not be effective.

Most studies on sugar labeling have been conducted in Western dietary countries with high-sugar diets, which is lacking in China. However, driven by rapid economic growth and drastic dietary changes, excessive sugar consumption in China is also becoming a notable problem. The average daily intake of added sugar (mainly sucrose, namely “white sugar” and “brown sugar”, etc.) in China is about 30 g, while the WTO and Chinese dietary guidelines recommend an intake of less than 25 g [6]. Overconsumption of added sugars can lead to a high public health burden. Medical literature shows that consumption of added sugars is strongly associated with obesity, type 2 diabetes, and other related diseases [7]. Currently, 11.2% of Chinese adults had diabetes in 2020, an increase of 10.53% since 1980 [8], higher than the global diabetes rate of 10.5% [9]. The overweight rate has also increased from 26.6% in 1993 to 41.3% in 2015 [10].

To address this situation, the introduction of sugar labeling on packaged foods in China is one of the necessary measures. In the “Healthy China Action (2019–2030)”, the Chinese government proposed the mandatory labeling of sugars such as sucrose and encouraged the use of claims for sugar-reduced foods [6]. Compared to the WHO guideline to control the intake of free sugars, which includes sugars added by the manufacturer, cook, or consumer, and sugars naturally present in honey, syrups, fruit juices, and fruit juice concentrates [11], the Chinese guideline focused more on added sugars in free sugars, especially sucrose. It is not mandatory for the whole industry at present; mainly, companies voluntarily post relevant information, which is worth further research.

This study is the first to evaluate the effect of sucrose grade labels in China, including “regular sucrose”, “low sucrose”, “no sucrose”, and “no sucrose with sugar substitutes” (in this study, we used “sucrose” to refer specifically to the added sugar in yogurt. It is important to note that traditional yogurt is made from milk and naturally contains lactose, a type of sugar. Our study focuses on added sugars, so we use “sucrose” to distinguish between naturally occurring lactose and added sucrose). The last three labels can be categorized into two main ways for food companies to produce sugar-reduced foods: by directly reducing sucrose or by using substitutes instead [6]. Consumer preference for these two options is important for retailers and food manufacturers. Therefore, yogurt was chosen as the subject of our study. First, compared to most sugar-sweetened beverages (SSBs), sucrose-reduced yogurt is more commonly produced in these two ways [12] (e.g., diet cola tends to use sugar substitutes, while fruit juice is generally reduced directly). Second, apart from table sugar (28.2%), sugary yogurt (21.9%) is the food with the highest contribution to sugar intake among urban Chinese [13], which is easily overlooked due to its health halo.

A two-stage experiment was designed to investigate consumer response to sucrose graded labels. The first stage focused on eliciting consumers’ willingness to pay (WTP) for sucrose graded labels. The second stage focused on their subsequent behavior in the absence of labels, specifically whether they compensated or maintained their sugar intake. This study is intended to address some of the concerns raised by some academics about compensatory eating and to provide additional valuable experimental evidence for policymakers to consider when evaluating the effectiveness of sugar labeling and even other dietary intervention strategies.

The rest of this paper is organized as follows. In section two, we discuss the existing research about sugar labeling and compensation and present our research hypotheses. Section three introduces the research method. Section four presents the empirical results. Section five presents policy implications and conclusions.

## 2. Literature Review

Nudge policies are gaining popularity worldwide as a means of influencing individuals’ decision-making processes by altering their potential responses to available options [14]. These policies are often used to promote healthy eating habits and can be classified as nudges that target cognition, affect, or behavior based on mental activity [15].

Sugar labeling is a common example of a cognitive nudge, which informs consumers about the sugar content or grade of a product to reduce excessive sugar intake [16]. Previous studies have mainly focused on traditional labeling, such as warning labels or sugar content information for high-sugar foods and beverages. These labels have been found to make consumers aware of the health risks of such products and help them reduce their consumption of high-sugar products [17,18,19].

Few studies have examined the labeling of sugar-reduced foods, such as “low-sugar”, “no-sugar” or “sugar substitute” [20]. It is worth noting that these labels not only represent different sugar content but also taste with different sweetness. Taste hedonics have a significant impact on food preferences and choices [21]. Consumers may prefer unhealthy products, despite recognizing their costs, because the resulting pleasure and utility may outweigh their concerns about potential health problems [22]. Therefore, despite previous studies showing that health and nutritional benefits have become an important factor in consumers’ food choices [23,24,25], it remains unknown whether consumers are willing to pay for labeled sugar-reduced products.

It is also important to consider the potential for spillover effects when designing policies to guide sugar consumption. For example, even after being influenced by labeling interventions, consumers who choose sugar-reduced foods may still consume other sugary snacks to compensate [5]. One possible explanation for this is that changing unhealthy behaviors through policy interventions can be challenging because consumers often persist in their dietary habits [26]. Diet acts as a memory, and once people’s eating habits are formed, they are often maintained for life [27]. In addition, dieters appear to have a more hedonic food orientation and tend to make palatable food choices unconsciously [28]. Even with conscious control, lack of knowledge makes it difficult for consumers to estimate added sugar content when faced with certain unlabeled foods [4].

In particular, sugar, unlike ordinary foods, is a substance that releases opioids and dopamine, and thus might be expected to have addictive potential [29]. Frequent exposure to high levels of sugar may enhance neural reward responses to palatable foods, leading to a dependence on consumption that is similar to substances of abuse and therefore difficult to stop [30,31]. After a period of sugar withdrawal, some people give in to a craving and end up consuming more sugar than they normally would. In all groups, young people are highly influenced by the food environment, and food addiction is more prevalent in the age group between 18 and 29 age group [32]. A study of a sample of college students found that more participants in the food addiction group reported that they should eat less sugary foods (96.7%) than those in the food addiction group without food addiction problems (55.5%). Participants who reported that they should eat less sugary foods were more likely to be in the food addiction group [33].

However, such spillover effects are rarely considered in studies of nudge policies. Most studies have focused only on examining the effects of nudge interventions on initial behavior change, without considering the issue of subsequent compensatory behavior. Downs et al. studied calorie labeling and suspected that those who consumed fewer calories as a result of the calorie labeling might later eat more at dinner [34]. Despite this concern among some scholars, no empirical studies have been conducted. In contrast to nudge policies, spillovers have been considered for the sugar tax, another intervention strategy for sugar. A related study found that a sugar tax may reduce consumption of SSBs, but cross-border shopping and switching to tax-exempt SSBs may mitigate this effect [35].

Therefore, using sugar labeling as a proxy for nudge policy, this study examines Chinese consumers’ preference for yogurt with sugar labeling and toast without sugar labeling to assess the consistency of consumers’ sugar control behavior. It contributes not only to the literature on the labeling of sugar-reduced foods but also to the spillover effects of the nudge policy.

## 3. Materials and Methods

### 3.1. Survey Design

**Figure 1:**
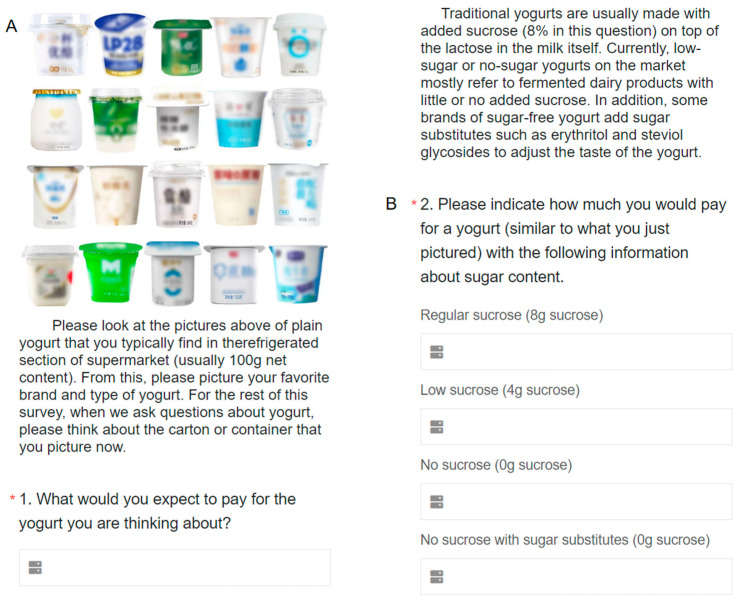
Pictures of yogurt consumers usually buy in grocery stores (**A**). Example for four sucrose grade labels (**B**). * It comes with the questionnaire and means that this question must be answered.

To investigate the impact of sucrose grade labels on consumer behavior, this study focused on yogurt, which is the second largest source of sugar intake for urban Chinese [13]. Four sucrose grade labels were selected to represent different sucrose contents: “regular sucrose”, “low sucrose”, “no sucrose”, and “no sucrose with sugar substitutes”. To minimize the potential bias caused by regional dietary culture differences, the specific gram amount of sucrose was indicated after each label (Figure 1). The sucrose concentrations were determined based on three levels: 8%, 4%, and 0% added sugar, representing regular, low, and no sucrose levels, respectively. Traditional yogurt typically contains an additional 5% to 10% sucrose [12,36], hence the selection of 8% as the regular level. The 4% and 0% concentrations were chosen in accordance with the GB 28050-2011 National Food Safety Standard General Rules for Nutrition Labeling of Prepackaged Foods in China (the requirements for nutrient content claim showed that products with “sugar-free or sugar excluded” claim should be ≤0.5 g/100 g (solid) or 100 mL (liquid), and products with “low sugar” claim should be ≤5 g/100 g (solid) or 100 mL (liquid)). The purposeful blurring of the yogurt images in Figure 1 is to eliminate the potential influence of brand recognition on the participants’ assessments.

**Figure 2:**
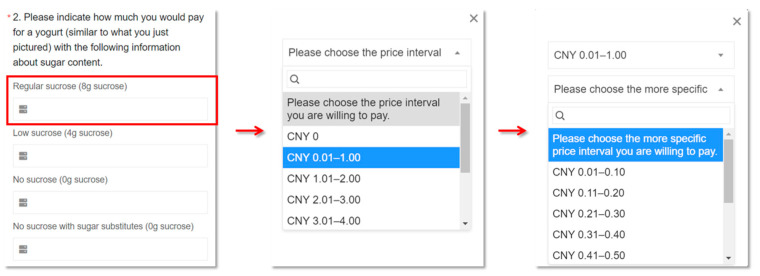
Example for payment card contingent valuation question with CNY 1.00 and CNY 0.10 price interval. * It comes with the questionnaire and means that this question must be answered.

The contingent valuation method (CVM) using payment cards was employed to assess consumers’ willingness to pay (WTP) for yogurt with different sucrose grade labels. This method was chosen due to its ability to address issues related to zero observations and initial bid numbers that are present in other CVM approaches [37]. In this study, hypothetical products were represented by 100 g of plain yogurt, which is commonly found in the refrigerated section of supermarkets. Respondents were asked to select a price interval that best represented their WTP for each yogurt. Considering that the average price of yogurt in China is CNY 2.08 (when the survey was launched on 1 February 2023, the central parity rate of China’s foreign exchange market was CNY 6.7492 for USD 1 and CNY 7.3318 for EUR 1) per 100 g [38] and that sucrose-reduced yogurt often commands a higher price premium in the market, the price interval was set from CNY 0.00 to CNY 8.00. The payment card design involved presenting respondents with initial price intervals of CNY 1.00, followed by subsequent intervals based on their initial choice to narrow the range down to CNY 0.10 intervals [39] (Figure 2).

**Figure 3:**
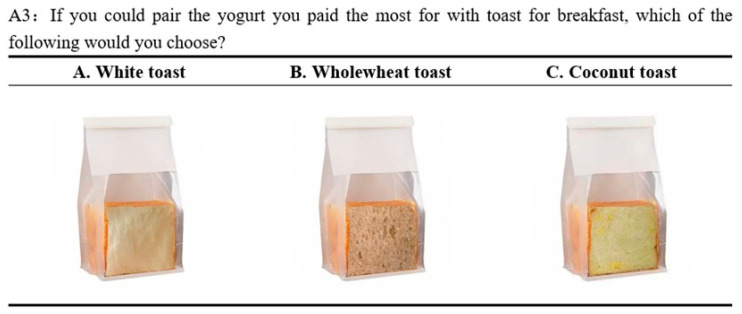
Example for three types of unlabeled toast.

In the second stage of the experiments, an examination was conducted to determine whether there was any compensatory eating behavior following the selection of sucrose-reduced yogurt. Participants who indicated the highest willingness to pay (WTP) for any of the four yogurt options were then presented with a choice of pairing that yogurt with toast for breakfast. The available toast options included wholewheat, white, and coconut toast (Figure 3). It is widely recognized that wholewheat toast is a healthier choice with lower sugar content compared to white toast or coconut toast [40]. Consequently, if a participant opted for wholewheat toast after expressing the highest WTP for any of the “low sucrose”, “no sucrose”, or “no sucrose with sugar substitutes” yogurts, it was inferred that they exhibited consistency in their sugar control behavior.

To test the influence on consumers’ sugar intake, this study considered the following variables: sociodemographic, consumption and lifestyle habits, diet-related health consciousness, and attitudes and knowledge about sugar reduction. Sociodemographic variables included age, gender, monthly living expenses, BMI, and household registration. Consumption habits were assessed by the frequency of yogurt consumption in the past week. Lifestyle questions included frequency of physical activities, smoking, alcohol consumption, diabetes, dieting, or weight loss. Diet-related health consciousness (Cronbach’s α = 0.811) and attitudes toward sugar reduction (Cronbach’s α = 0.930) were measured by a series of five-point Likert scale questions [16,41]. Knowledge of sugar reduction was measured by five true/false questions [41].

### 3.2. Data Collection

This experiment was conducted at a university in February 2023. University students were chosen as the sample because of the following characteristics: First, compared to others, university students have more autonomy and independence in food choices [42], which may reduce bias due to family-level influences (e.g., consider parents or children when choosing) on the statistics. Second, compared to their elders, the younger generation is more influenced by the westernization of diets, and their sugar consumption is higher than the national average [43,44], which is of particular concern and deserves more attention. In addition, these findings occur during “emerging adulthood” [45], and these behaviors may persist into later adulthood [46,47]. Studying their current consumption habits and behaviors will provide policy insights for reducing sugar consumption in response to healthy eating in the future. Therefore, studying behavioral health interventions during this period is important to predict future trends and reduce the risk of adverse health outcomes later in life [48].

To collect data, 2000 invitation cards were randomly distributed throughout the school, offering students the opportunity to complete the questionnaire by scanning a QR code using WeChat, as described by [49]. In order to enhance data quality, a cheap talk script was used before conducting the contingent valuation method (CVM) [50]. This script was designed to mitigate hypothetical bias by giving the following instructions to participants: “Results from previous surveys have shown that people’s willingness to pay is higher than their actual willingness to pay for the product in the store, which is known as ‘hypothetical bias’. To avoid ‘hypothetical bias’, we ask you to answer each of the following preference questions exactly as you would if you were in a real store and had to pay for your choice. And remember that if you buy a product, you will have less money available for other purchases. Your choice is very important to the study and your cooperation is appreciated”. In addition, a validation question with a predetermined correct response was included in accordance with the methodology employed [51]. The responses that deviated from the given answer were considered unreliable and excluded from the analysis. Ultimately, a total of 953 students participated in the study, with 892 responses deemed valid for further analysis. All statistical analyses were performed using Stata software (17.0, Stata Corp, College Station, TX, USA).

### 3.3. Econometric Model

Since the payment card choices are in the form of price intervals, we assume the true value of WTP by calculating the midpoint of the interval [37]. Specifically, respondents’ WTP for yogurt is estimated as follows:

(1) $$E\left(WTP\right)=\sum P_{i}*W_{i}$$

where $\;W_{i}$ represents the ith price interval chosen by the respondent, and $P_{i}$ is the probability that this respondent chooses the price interval $W_{i}$.

After estimating WTP, due to the lack of homogeneity of variance in the data, Kruskal–Wallis tests are used to examine whether there exists a significant difference in consumers’ WTP among the four sucrose grade labels.

In addition, to investigate the effect of the influencing factors on WTP, we use the ordinary least squares (OLS) estimation and perform robustness tests by using the Tobit model. The OLS model is set up as follows:

(2) $$y_{i}^{\;}=\alpha +X_{i}\beta +\mu_{i}\;\;$$

where $y_{i}$ represents the WTP of consumer i, and $X_{i}$ contains explanatory variables such as attitudes, knowledge, habits, and demographic characteristics. Thus, the estimated β represents the effect of $X_{i}$ on $y_{i}^{\;}$.

When the variable to be explained is binomial, the logit model is widely used for analysis [52]. Thus, we use the logit model to investigate the factors that influence the consistency of sugar control behaviors. The distribution probability of binary variables is as follows:

(3) $$\left\{\begin{array}{c} P\left(y=1|x\right)=F\left(x,\beta \right)\\ P\left(y=0|x\right)=1-F\left(x,\beta \right) \end{array}\right.$$

If the cumulative distribution function $F\left(x,\beta \right)$ is a logistic distribution, the logit model is built as follows:

(4) $$P\left(y=1|x\right)=F\left(x,\beta \right)=\Lambda \left(x^{\prime }\beta \right)=\frac{exp\left(x^{\prime }\beta \right)}{1+exp\left(x^{\prime }\beta \right)}$$

After MLE estimation, the probability density of the ith observation value is as follows:

(5) $$f\left(y_{i}|x_{i},\beta \right)=\left\{\begin{array}{c} \Lambda \left(x^{\prime }\beta \right)\;\;\;\left(y_{i}=1\right)\\ 1-\Lambda \left(x^{\prime }\beta \right)\;\;\;\left(y_{i}=0\right) \end{array}\right.$$

where $y_{i}$ indicates whether the ith respondent is consistent with sugar control, and the remaining variables are the same as in the OLS model.

## 4. Results

### 4.1. Data and Descriptive Statistics

Table 1 provides a statistical description of our data. Specifically, the sample included 18–24-year-olds, with 43.95% male and 56.05% female, and the gender ratio was similar to that of students at colleges and universities nationwide (50% for each gender) [53]. In terms of monthly living expenses, 8.30% spent CNY 1000 or less, 34.64% spent CNY 1001–1500, 40.13% spent CNY 1501–2000, 14.35% spent CNY 2001–3000, and 2.58% spent CNY 3001 or more; the distribution of university students in China is 22.34%, 39.78%, 22.45%, 10.42%, and 5.01%, respectively [54]. In terms of BMI, 585 (65.58%) respondents were of normal weight, 108 (12.11%) were overweight, and 51 (5.72%) were obese. Over half of the sample (56.73%) were from urban areas, and the remaining 43.27% were from rural areas. In addition, 230 (25.78%) respondents were on a weight loss diet, and 10 (1.12%) respondents were diabetic.

In terms of consumption and lifestyle, most of the respondents consumed yogurt 1–2 times in the past week, accounting for 50.45% of our sample. The majority of respondents exercised 1–2 times or not at all in the past week, 76.79% in total, with a relatively low frequency of exercise. A total of 95.07% of the students never smoked. And the frequency of alcohol consumption was mainly concentrated in “Never” (57.40%) and “Once in a while” (41.93%), which is relatively healthy overall. 

As for diet-related health consciousness, the average score was about 4.112, indicating that the respondents have a relatively high dietary health awareness. The mean scores for knowledge and attitude of sugar control were 3.130 and 4.160, respectively. This indicates that the respondents have a positive attitude toward sugar control, but their related knowledge is still insufficient.

**Figure 4:**
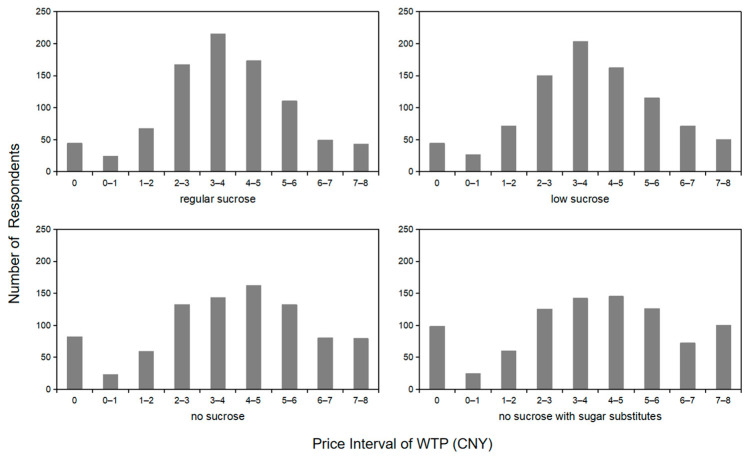
The frequency of WTP for different sucrose grade labels.

The frequency distribution of the respondents’ choices is shown in Figure 4. The respondents’ WTP modes for “regular sucrose” and “low sucrose” yogurt are in the range of CNY 3.00–4.00, and the WTP modes for “no sucrose” and “no sucrose with sugar substitutes” yogurt are in the range of CNY 4.00–5.00. Compared with “regular sucrose” and “low sucrose” yogurt, the distribution of two types of “no sucrose” yogurt is more in the range of CNY 0.00 and CNY 7.00–8.00; that is, they are not willing to purchase the two types of yogurt, or they are willing to pay the highest price. 

### 4.2. Statistics of WTP

Table 2 reports the WTP calculated using Equation (1). The respondents were willing to pay CNY 3.66, CNY 3.80, CNY 3.92, and CNY 3.92 for the “regular sucrose”, “low sucrose”, “no sucrose”, and “no sucrose with sugar substitutes” yogurts, respectively. The result indicates that consumers are willing to pay about 4%, 7%, and 7% more for “low sucrose”, “no sucrose”, and “no sucrose with sugar substitutes” yogurt compared to “regular sucrose” yogurt. This result is consistent with the prediction made in the previous study [38]. In addition, the results of Kruskal–Wallis tests show that consumers’ WTP differs significantly among these labels.

Furthermore, the results of the pairwise K-W test showed that consumers’ WTP for “low sucrose”, “no sucrose”, and “no sucrose with sugar substitutes” yogurt was significantly higher compared to “regular sucrose” yogurt (at the 10%, 1%, and 11% significance levels, respectively). Consumers’ WTP for “no sucrose” and “no sucrose with sugar substitutes” yogurt was significantly higher than for “low sucrose” yogurt. Meanwhile, there was no significant difference between WTP for “no sucrose” and “no sucrose with sugar substitutes” yogurt, indicating that consumers may value the reduction in sucrose more than the potential change in taste resulting from the use of sugar substitutes.

### 4.3. Influencing Factors of WTP

Table 3 reports the estimation results of WTP for different sucrose grade labels by using OLS.

The results of the study indicate that respondents’ socioeconomic characteristics play a significant role in their WTP for yogurts with different sucrose grade labels. Specifically, women had a lower WTP for “regular sucrose” yogurt, but a higher WTP for “no sucrose” and “no sucrose with sugar substitutes” yogurt compared to men. Moreover, BMI status also influenced WTP, with normal-weight students showing a lower WTP for “regular sucrose” yogurt, while overweight students showed a higher WTP for “no sucrose” yogurt. Students from rural areas exhibited a lower WTP for “no sucrose with sugar substitutes” yogurt. In addition, respondents’ WTP for all types of yogurts increased in tandem with their monthly living expenses.

The analysis also reveals associations between respondents’ lifestyle habits and their WTP for yogurts with different sucrose grade labels. Specifically, as the frequency of exercise increased, respondents’ WTP for both “regular sucrose” and “low sucrose” yogurt decreased. Students with diabetes had a significantly lower WTP for “low sucrose” yogurt. 

Moreover, respondents who were trying to lose weight showed a higher WTP for “low sucrose”, “no sucrose”, and “no sucrose with sugar substitutes” yogurts. The analysis demonstrated that when respondents were actively engaged in weight loss, their WTP for “low sucrose”, “no sucrose”, and “no sucrose with sugar substitutes” yogurts increased by CNY 0.26, CNY 0.47, and CNY 0.52, respectively. In addition, smoking frequency had a negative effect on respondents’ willingness to pay WTP for both “low sucrose” and “no sucrose” yogurts. 

### 4.4. Consistent Sugar Control Behavior and Influencing Factors

**Figure 5:**
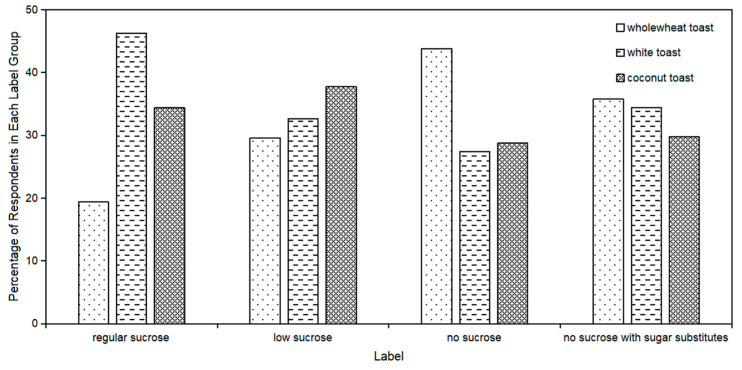
Proportion of toast preference among respondents with different yogurt preferences.

To investigate the potential compensatory behavior of consumers who opt for sucrose-reduced yogurt, 706 respondents were retained in the sample who showed strict preferences for sucrose grade labels in the last stage, as they were only willing to pay the highest WTP for one of the four labels. The number of respondents in the regular sucrose, low sucrose, no sucrose, and no sucrose with sugar substitutes groups were 160, 98, 146, and 302 respectively. Compared to consumers of “regular sucrose” yogurt, a higher proportion of consumers of “low sucrose”, “no sucrose”, and “no sucrose with sugar substitutes” yogurt opted for wholewheat toast, while the proportion choosing white toast decreased (Figure 5). 

The correlation between consumer preference for yogurts with different sucrose grade labels and toast has been shown in Table 4. The preference for “regular sucrose” yogurt was positively associated with the preference for white toast, whereas it was negatively associated with the preference for wholewheat toast. On the other hand, the preference for “no sucrose” yogurt was negatively associated with the preference for white toast, but positively associated with a preference for wholewheat toast. 

Table 5 presents the results of a logit model that examines the factors that influence consistency in sugar control behavior. If a respondent chose wholewheat toast after indicating the highest WTP for one of the “low sucrose”, “no sucrose”, and “no sucrose with sugar substitutes” yogurts, they were assumed to maintain their sugar control behaviors. Consistent with the findings in Table 3, the coefficient for gender was statistically significant, indicating that female consumers exhibit greater consistency in reducing sugar intake. Moreover, normal-weight and overweight individuals were more likely to maintain consistent sugar control compared to underweight individuals. Exercise frequency showed a negative relationship with sugar control behavior. Furthermore, the coefficient for those trying to lose weight was statistically significant. The awareness of healthy eating habits and knowledge of sugar reduction play significant roles. Consumers who were more concerned about healthy eating and more knowledgeable about reducing sugar intake were more likely to maintain consistent sugar control practices.

## 5. Discussion

Previous studies have focused on traditional sugar labeling, such as warning signals or sugar content information for high-sugar products [55]. Few studies have examined the claims on sugar-reduced foods. This study demonstrates that Chinese university students are willing to pay a different premium for yogurts with sucrose reduction labels, which is consistent with the prediction made in the previous study [38]. The premium increases with the degree of sucrose reduction. No significant disparity is observed in the WTP for yogurt labeled as “no sucrose” versus “no sucrose with sugar substitutes”, but the people who like the two yogurts are slightly different. In addition, the respondents are willing to pay CNY 3.66 for “regular sucrose” than the average yogurt price calculated by the previous study of 2.08 CNY/100 g. This may be because the latter price is calculated by dividing the total expenditure by the total volume for all yogurt sizes and includes larger portions of yogurt [38]. In addition, our study was conducted among young people, who may now have a higher willingness to pay for a variety of yogurts than other age groups [56].

Another interesting finding is that the majority of consumers who choose sucrose-reduced yogurts continue to select toast with lower sugar content, demonstrating their commitment to maintaining their sugar control behavior. They are able to remain consistent with their first choice and do not show compensation for sugary foods, even though they were not labeled. In contrast, respondents who prefer “regular sucrose” yogurt also prefer white toast while disliking wholewheat toast, perhaps because these respondents prefer the hedonic pleasure of food sensations [57], or because they consider toast to be a healthy food in itself due to their lack of knowledge compared to people who maintain their sugar control behavior (Table 5). Previous research has found that other interventions, such as limiting beverage size, may have the unintended consequence of increasing, rather than decreasing, soda consumption [58]. However, in this study, sucrose grade labels are effective in promoting sugar reduction, the intended health benefits of the labels are not lost, and they are not counterproductive in guiding healthy eating. This conclusion echoes concerns from previous research about compensatory eating following dietary interventions [5,34] and further supports the effectiveness of sugar labeling.

Consumer WTP for sucrose grade labels and whether to maintain sugar control behaviors varies across demographic groups. Our research shows that women have higher WTP for sucrose-reduced yogurts, which may be due to women’s greater emphasis on health concerns, while men may have higher energy needs [59,60]. Additional factors, such as exercise frequency and those who are dieting or losing weight, also positively influence WTP for sucrose-reduced yogurts, suggesting that individuals who prioritize physical activity may be more aware of their sugar and calorie intake, as well as their overall dietary health, and are more inclined to value and invest in healthier food options [16]. Rural respondents showed a negative preference for sugar substitutes, probably because these types of products are more likely to be promoted initially by new retail supermarkets than by traditional channels, and are therefore less common in rural areas than in urban areas [61]. In addition, the more frequently respondents smoked, the less they liked healthy yogurt, possibly because smokers may prioritize immediate gratification over health consciousness, resulting in a lower value placed on healthier food options. This finding is consistent with previous research that has also highlighted the negative impact of smoking on health-related decision-making [62,63]. Another explanation is that high cigarette exposure is associated with low taste perception at the tip of the tongue [64], and therefore, smokers may prefer palatable products. In terms of maintaining sugar control behavior, individuals who are trying to lose weight show greater consistency in sugar control, possibly because they are more aware of sugar content or calorie intake in unlabeled foods. Respondents who exercised more frequently were less likely to maintain sugar control, and one possible explanation is that regular exercisers may require higher levels of sugar in their diet to provide energy.

The findings of this study will provide valuable insights for food manufacturers. Sugar-reduced products are promising among young people in China. Sugar reduction claims can be an effective marketing strategy for food companies to differentiate products and attract consumers. Companies that reformulate food products can play an important role in helping consumers eat healthier. Not only is it required by public health policy [65], but our study shows that it is also an effective way for companies to improve their competitiveness in the marketplace. In addition, women, regular exercisers, and people who are trying to lose weight may be the target market for sugar-reduced products. 

The results will also provide policymakers with insights into the effectiveness of sugar labeling. The westernization of diets in China has led to a significant increase in the prevalence of obesity, type 2 diabetes, and other related diseases among young individuals [66,67]. Projections indicate that by 2030, the healthcare costs associated with overweight and obesity in China will account for approximately 22% of the country’s total medical expenditures [68]. Consequently, there is an urgent and imperative need to guide consumers, particularly young people, in reducing their consumption of added sugars. This study highlights that interventions involving sugar labeling not only serve as a short-term guide for reducing sugar consumption but also have the potential to induce long-term behavioral changes. These findings hold significant implications for public health policies on a global level. It is worth noting that the guiding effectiveness of different sugar reduction policies varies [16]. In this study, the effectiveness of sugar labeling was examined using sucrose grade labels as an example, and the sustainability of other interventions remains to be tested.

The design of effective strategies and interventions to reduce the high prevalence of obesity and its comorbidities should also take into account the characteristics of the population. Male consumers may require targeted health education to reduce sugar intake and related compensatory behaviors. Meanwhile, for consumers who are not dieting, policymakers could focus on increasing the accessibility and appeal of healthy food options to guide healthier choices. For those who are obese (with a high BMI), personalized and long-term support such as professional health counseling and diet tracking tools should be provided to help them better manage their diets. In addition, consumers with higher levels of dietary health awareness and knowledge about sugar reduction are more likely to maintain their sugar control behaviors. In response, the government can increase advertising and education to make people aware of the risks of excessive sugar consumption [69]. 

This study has several limitations. One of them is the research sample. Our analysis focused on a specific demographic; the responses of young people without the same education and even the broader population are also worth testing in the future. The survey design can also be improved. Regarding the images used in the experiment, it is recommended that future research adopt a consistent approach to product presentation (e.g., using both mock products) across all phases of the experiment to eliminate the potential confounding effects of product presentation differences. In terms of compensatory behavior, this study only took toast as an example in the second stage. Further research, especially in controlled trials and real-life situations, should investigate whether compensatory eating occurs during the next meal and whether there are differences in compensatory behavior between different products (e.g., between hedonic and virtue products). In addition, this study mainly focused on the impact of sucrose grade labels and consumer preference. In May 2023, the WHO issued new guidelines advising against the use of non-sugar sweeteners that have potential adverse effects with long-term use [70]. Future research may consider the risk of sugar substitutes. 

## Figures and Tables

**Table 1:** Descriptive statistics of the sample.

Variable		Proportion/Mean Value
Gender	Male	43.95%
Age	Mean value	21.03
Expense (CNY per month)	1000 or less	8.30%
	1001–1500	34.64%
	1501–2000	40.13%
	2001–3000	14.35%
	3001 or above	2.58%
BMI	Underweight (BMI < 18.5)	16.59%
	Normal (18.5 ≤ BMI < 24)	65.58%
	Overweight (24 ≤ BMI < 28)	12.11%
	Obese (28 ≤ BMI)	5.72%
Household	Urban	56.73%
Diabetes	True	1.12%
Diet or lose weight	True	25.78%
Yogurt (in the past week)	0 times	24.89%
	1–2 times	50.45%
	3–4 times	18.83%
	5–6 times	4.48%
	7 times or above	1.35%
Exercise (in the past week)	0 times	30.38%
	1–2 times	46.41%
	3–4 times	15.36%
	5–6 times	5.94%
	7 times or above	1.91%
Smoke	Never	95.07%
	Once in a while	3.36%
	Every day	1.57%
Drink	Never	57.40%
	Once in a while	41.93%
	Every day	0.67%
Diet-related health consciousness	Mean value	4.112
Knowledge of sugar reduction	Mean value	3.130
Attitude to sugar reduction	Mean value	4.160

**Table 2:** Mean WTP for sucrose grade labels.

Label	WTP (CNY)	
	Mean	S.D.
Regular sucrose	3.658	1.863
Low sucrose	3.800	1.965
No sucrose	3.924	2.213
No sucrose with sugar substitutes	3.922	2.342

**Table 3:** Estimation of WTP for different sucrose grade labels.

	(1)	(2)	(3)	(4)
Variables	Regular Sucrose	Low Sucrose	No Sucrose	No Sucrose with Sugar Substitutes
Female	−0.292 **	0.166	0.572 ***	0.590 ***
	(0.140)	(0.147)	(0.164)	(0.174)
Age	−0.055	−0.077 *	0.023	−0.008
	(0.038)	(0.040)	(0.045)	(0.048)
Expense _1001–1500_	0.179	−0.004	0.187	0.174
	(0.245)	(0.257)	(0.287)	(0.305)
Expense _1501–2000_	0.333	0.291	0.305	0.575 *
	(0.246)	(0.259)	(0.288)	(0.306)
Expense _2001–3000_	0.642 **	0.360	0.559 *	0.756 **
	(0.281)	(0.295)	(0.329)	(0.349)
Expense _3001 or above_	0.811 *	1.288 ***	1.453 ***	0.771
	(0.452)	(0.475)	(0.529)	(0.562)
BMI _normal_	−0.294 *	0.092	0.126	0.127
	(0.174)	(0.183)	(0.204)	(0.216)
BMI _overweight_	−0.082	0.407	0.560 *	0.110
	(0.246)	(0.258)	(0.288)	(0.306)
BMI _obese_	0.284	0.282	−0.065	0.203
	(0.321)	(0.337)	(0.376)	(0.399)
Rural	−0.095	−0.153	−0.193	−0.358 **
	(0.131)	(0.138)	(0.154)	(0.164)
Yogurt _1–2 times_	0.305 **	0.389 **	0.381 **	0.380 **
	(0.155)	(0.163)	(0.182)	(0.193)
Yogurt _3–4 times_	0.098	0.257	0.429 *	0.344
	(0.197)	(0.207)	(0.231)	(0.245)
Yogurt _5–6 times_	0.220	0.392	−0.117	−0.210
	(0.328)	(0.344)	(0.384)	(0.408)
Yogurt _7 times or above_	−0.610	−0.427	−0.614	0.267
	(0.561)	(0.589)	(0.657)	(0.698)
Exercise _1–2 times_	−0.266 *	−0.316 **	−0.070	−0.075
	(0.149)	(0.156)	(0.174)	(0.185)
Exercise _3–4 times_	−0.437 **	−0.238	−0.084	0.004
	(0.202)	(0.213)	(0.237)	(0.252)
Exercise _5–6 times_	−0.701 **	−0.695 **	0.029	−0.136
	(0.292)	(0.307)	(0.342)	(0.363)
Exercise _7 times or above_	−0.156	−0.851 *	0.311	0.463
	(0.486)	(0.511)	(0.569)	(0.605)
Diabetes	−0.982	−1.071 *	−0.587	−0.337
	(0.611)	(0.642)	(0.716)	(0.761)
Diet or lose weight	−0.054	0.261 *	0.465 ***	0.519 ***
	(0.150)	(0.158)	(0.176)	(0.187)
Smoke _once in a while_	0.026	0.130	−0.113	−0.095
	(0.365)	(0.384)	(0.428)	(0.455)
Smoke _every day_	−0.912 *	−1.278 **	−1.578 ***	−0.880
	(0.519)	(0.545)	(0.608)	(0.646)
Drink _once in a while_	−0.168	0.050	0.137	0.173
	(0.132)	(0.139)	(0.155)	(0.165)
Drink _every day_	0.221	−0.166	−0.150	0.847
	(0.779)	(0.819)	(0.913)	(0.970)
Diet-related health consciousness	−0.052	−0.045	−0.057	−0.139
	(0.099)	(0.104)	(0.116)	(0.123)
Knowledge of sugar reduction	−0.018	0.037	0.081	0.082
	(0.053)	(0.056)	(0.062)	(0.066)
Attitude to sugar reduction	−0.102	0.026	0.075	0.062
	(0.090)	(0.095)	(0.106)	(0.113)
Constant	5.757 ***	4.953 ***	2.057 *	3.040 ***
	(0.923)	(0.970)	(1.081)	(1.149)
Number of observations	892	892	892	892
R-squared	0.053	0.060	0.079	0.071

Note: ***, **, and * represent statistical significance at the 1%, 5%, and 10% levels, respectively, and standard errors are in parentheses.

**Table 4:** Correlation between sucrose grade label and toast preference.

Label	White Toast	Wholewheat Toast	Coconut Toast
Regular sucrose	0.123 ***	−0.155 ***	0.031
Low sucrose	−0.023	−0.028	0.052
No sucrose	−0.086 **	0.119 ***	−0.032
No sucrose with sugar substitutes	−0.018	0.053	−0.036

Note: ***, ** represent statistical significance at the 1% and 5% levels, respectively.

**Table 5:** Estimation of factors influencing consistency in sugar control behavior.

Variables	Coefficients	Standard Error
Female	0.496 **	0.207
Age	0.024	0.057
Expense _1001–1500_	−0.378	0.358
Expense _1501–2000_	−0.378	0.360
Expense _2001–3000_	−0.710 *	0.419
Expense _3001 or above_	1.739 **	0.757
BMI _normal_	0.566 **	0.269
BMI _overweight_	0.758 **	0.354
BMI _obese_	0.088	0.495
Rural	0.050	0.190
Yogurt _1–2 times_	0.078	0.227
Yogurt _3–4 times_	0.362	0.289
Yogurt _5–6 times_	−0.009	0.515
Yogurt _7 times or above_	0.453	0.898
Exercise _1–2 times_	−0.209	0.213
Exercise _3–4 times_	−0.282	0.299
Exercise _5–6 times_	−0.217	0.407
Exercise _7 times or above_	−2.323 **	1.095
Diabetes	−1.144	1.151
Diet or lose weight	0.924 ***	0.204
Smoke _once in a while_	0.053	0.529
Smoke _every day_	−0.284	0.848
Drink _once in a while_	0.460 **	0.192
Drink _every day_	1.714	1.324
Diet-related health consciousness	0.387 **	0.157
Knowledge of sugar reduction	0.135 *	0.080
Attitude to sugar reduction	−0.009	0.131
Constant	−4.266 ***	1.396
Observations	706	
LR statistic	85.10	
Pseudo R-squared	0.101	

Note: ***, **, and * represent statistical significance at the 1%, 5%, and 10% levels, respectively, and standard errors are in parentheses.

## Data Availability

The data presented in this study are available upon request from the corresponding author. The data are not publicly available due to privacy restrictions.

## References

[1] Diaz J., Sanchez A., Diez-Canseco F., Miranda J.J., Popkin B.M. (2023). Employment and wage effects of sugar-sweetened beverage taxes and front-of-package warning label regulations on the food and beverage industry: Evidence from Peru. Food Policy.

[2] Leung C.W., Wolfson J.A., Hsu R., Soster K., Mangan S., Falbe J. (2020). Warning Labels Reduce Sugar-Sweetened Beverage Intake among College Students. J. Nutr..

[3] Jindahra P., Phumpradab P. (2023). Label copresence for healthier choices: How sugar content per daily limit and sugar warning labels balance out the health halos of nutrient-content claim. Food Qual. Prefer..

[4] Weaver D., Finke M. (2003). The relationship between the use of sugar content information on nutrition labels and the consumption of added sugars. Food Policy.

[5] Gibson S., Ashwell M., Arthur J., Bagley L., Lennox A., Rogers P.J., Stanner S. (2017). What can the food and drink industry do to help achieve the 5% free sugars goal?. Perspect. Public Health.

[6] The State Council of China (2019). Healthy China Action (2019–2030).

[7] Sainsbury E., Magnusson R., Thow A.-M., Colagiuri S. (2020). Explaining resistance to regulatory interventions to prevent obesity and improve nutrition: A case-study of a sugar-sweetened beverages tax in Australia. Food Policy.

[8] Chinese Diabetes Society (2021). Guideline for the prevention and treatment of type 2 diabetes mellitus in China (2020 edition) (Part 1). Chin. J. Pract. Intern. Med..

[9] IDF IDF Diabetes Atlas. https://diabetesatlas.org/data/en/country/42/cn.html.

[10] Ma S., Xi B., Yang L., Sun J., Zhao M., Bovet P. (2021). Trends in the prevalence of overweight, obesity, and abdominal obesity among Chinese adults between 1993 and 2015. Int. J. Obes..

[11] WHO (2015). Guideline: Sugars Intake for Adults And Children.

[12] Wan Z., Khubber S., Dwivedi M., Misra N.N. (2020). Strategies for lowering the added sugar in yogurts. Food Chem..

[13] Chinese Nutrition Society (2022). The 2022 Scientific Research Report on Dietary Guidelines for Chinese Residents.

[14] Matjasko J.L., Cawley J.H., Baker-Goering M.M., Yokum D.V. (2016). Applying Behavioral Economics to Public Health Policy: Illustrative Examples and Promising Directions. Am. J. Prev. Med..

[15] Cadario R., Chandon P. (2019). Viewpoint: Effectiveness or consumer acceptance? Tradeoffs in selecting healthy eating nudges. Food Policy.

[16] Hagmann D., Siegrist M., Hartmann C. (2018). Taxes, labels, or nudges? Public acceptance of various interventions designed to reduce sugar intake. Food Policy.

[17] Taillie L.S., Higgins I.C.A., Lazard A.J., Miles D.R., Blitstein J.L., Hall M.G. (2022). Do sugar warning labels influence parents’ selection of a labeled snack for their children? A randomized trial in a virtual convenience store. Appetite.

[18] Musicus A.A., Moran A.J., Lawman H.G., Roberto C.A. (2019). Online randomized controlled trials of testaurant sodium warning labels. Am. J. Prev. Med..

[19] Khandpur N., Rimm E.B., Moran A.J. (2020). The influence of the New US nutrition facts label on consumer perceptions and understanding of added sugars: A randomized controlled experiment. J. Acad. Nutr. Diet..

[20] Prada M., Saraiva M., Sério A., Coelho S., Godinho C.A., Garrido M.V. (2021). The Impact of Sugar-Related Claims on Perceived Healthfulness, Caloric Value and Expected Taste of Food Products. Food Qual. Prefer..

[21] Armitage R.M., Iatridi V., Thanh V.C., Yeomans M.R. (2023). Phenotypic differences in taste hedonics: The effects of sweet liking. Food Qual. Prefer..

[22] Becker G.S., Murphy K.M. (1988). A theory of rational addiction. J. Political Econ..

[23] Kim K., Cheong Y., Zheng L. (2009). The current practices in food advertising. Int. J. Advert..

[24] Hong X., Li C., Wang L., Gao Z., Wang M., Zhang H., Monahan F.J. (2022). The Effects of Nutrition and Health Claim Information on Consumers’ Sensory Preferences and Willingness to Pay. Foods.

[25] Tian Y., Zhu H., Zhang L., Chen H. (2022). Consumer Preference for Nutritionally Fortified Eggs and Impact of Health Benefit Information. Foods.

[26] Riet J.V., Sijtsema S.J., Dagevos H., De Bruijn G. (2011). The importance of habits in eating behaviour. An overview and recommendations for future research. Appetite.

[27] Verplanken B., Aarts H. (1999). Habit, Attitude, and Planned Behaviour: Is Habit an Empty Construct or an Interesting Case of Goal-directed Automaticity? Eur. Rev. Soc. Psychol..

[28] Luomala H.T., Hellén K., Jokitalo M. (2018). Dieting, priming, food meanings and (un)healthy choices: When shoppers fall for pleasure. J. Retail. Consum. Serv..

[29] Avena N.M., Rada P., Hoebel B.G. (2008). Evidence for sugar addiction: Behavioral and neurochemical effects of intermittent, excessive sugar intake. Neurosci. Biobehav. Rev..

[30] DiNicolantonio J.J., O’Keefe J.H., Wilson W.L. (2018). Sugar addiction: Is it real? A narrative review. Br. J. Sports Med..

[31] Thanarajah S.E., DiFeliceantonio A.G., Albus K., Kuzmanovic B., Rigoux L., Iglesias S., Hanßen R., Schlamann M., Cornely O.A., Brüning J.C. (2023). Habitual daily intake of a sweet and fatty snack modulates reward processing in humans. Cell Metab..

[32] Hauck C., Weiss A., Schulte E.M., Meule A., Ellrott T. (2017). Prevalence of ‘Food Addiction’ as Measured with the Yale Food Addiction Scale 2.0 in a Representative German Sample and Its Association with Sex, Age and Weight Categories. Obes. Facts.

[33] Romero-Blanco C., Hernandez-Martinez A., Parra-Fernandez M.L., Onieva-Zafra M.D., Prado-Laguna M., Rodriguez-Almagro J. (2021). Food Addiction and Lifestyle Habits among University Students. Nutrients.

[34] Downs J.S., Loewenstein G., Wisdom J. (2009). Strategies for Promoting Healthier Food Choices. Am. Econ. Rev..

[35] Zhang Q., McCluskey J.J., Gallardo R.K., Brady M.P. (2021). Avoidance behaviors circumventing the sugar-sweetened beverages tax. Food Policy.

[36] Chollet M., Gille D., Schmid A., Walther B., Piccinali P. (2013). Acceptance of sugar reduction in flavored yogurt. J. Dairy Sci..

[37] Yu X., Gao Z., Zeng Y. (2014). Willingness to pay for the “Green Food” in China. Food Policy.

[38] Chen B., Zhou Q., Zhang X. (2021). Product differentiation and brand building: A hedonic analysis of yogurt price in China. Int. Food Agribus. Manag. Rev..

[39] Hu Y., House L.A., Gao Z. (2023). Does Preferred Information Format Affect Consumers ‘ Willingness to Pay: A Case Study of Orange Juice Produced by Biotechnology. Foods.

[40] Van Kleef E., Seijdell K., Vingerhoeds M.H., De Wijk R.A., Van Trijp H.C.M. (2018). The effect of a default-based nudge on the choice of whole wheat bread. Appetite.

[41] Tang Q., Lin Q., Yang Q., Sun M., Liu H., Yang L. (2020). Knowledge, Attitude, and Practice of Adolescent Parents on Free Sugar and Influencing Factors about Recognition. Int. J. Environ. Res. Public Health.

[42] Wengreen H., Moncur C. (2009). Change in diet, physical activity, and body weight among young-adults during the transition from high school to college. Nutr. J..

[43] Xu X., Pu Y., Sharma M., Rao Y., Cai Y., Zhao Y. (2017). Predicting physical activity and healthy nutrition behaviors using social cognitive theory: Cross-sectional survey among undergraduate students in Chongqing, China. Int. J. Environ. Res. Public Health.

[44] Liu Q., Zhang X., Wang Z., Li L., Li Z., Xie Y. (2023). Investigation of added sugar intake among college students in Shijiazhuang City and analysis of influencing factors. Food Nutr. China.

[45] Arnett J. Emerging adulthood: The winding road from the late teens through the twenties. Oxford University Press: Cary, NC, USA, 2004; pp. 469–480.

[46] Nelson M.C., Story M., Larson N.I., Neumark-Sztainer D., Lytle L.A. (2008). Emerging adulthood and college-aged youth: An overlooked age for weight-related behavior change. Obesity.

[47] Bailey C.P., Sharma S., Economos C.D., Hennessy E., Simon C., Hatfield D.P. (2020). College campuses’ influence on student weight and related behaviours: A review of observational and intervention research. Obes. Sci. Pract..

[48] Winpenny E.M., Penney T.L., Corder K., White M., van Sluijs E. (2017). Changes in consumption of added sugars from age 13 to 30 years: A systematic review and meta-analysis of longitudinal studies. Obes. Rev..

[49] Quan S., Yu X., Zeng Y. (2017). A study of consumer preference for milk powder origin in china—A comparative analysis based on choice experiment and display preference data. J. Agrotech. Econ..

[50] Joseph F., Matthew R. (1996). Cheap Talk. J. Econ. Perspect..

[51] Gao Z.F., House L.A., Xie J. (2016). Online survey data quality and its implication for willingness-to-pay: A cross-country comparison. Can. J. Agric. Econ.-Rev. Can. D’agroeconomie.

[52] Atanu S., Alan L.H., Robert S. (1994). Adoption of emerging technologies under output uncertainty. Am. J. Agric. Econ..

[53] China National Bureau of Statistics (2022). Statistical Monitoring Report on the Program for the Development of Chinese Women (2021–2030) in 2022.

[54] XiaoGuo Research Institute 2021 College Students Consumer Behavior Insight Report.

[55] An R., Liu J., Liu R., Barker A.R., Figueroa R.B., McBride T.D. (2021). Impact of Sugar-Sweetened Beverage Warning Labels on Consumer Behaviors: A Systematic Review and Meta-Analysis. Am. J. Prev. Med..

[56] FBIF Food and Beverage Innovation More and More Expensive Yogurt, How to Prop up Young People’s “Yogurt Freedom”?. https://www.36kr.com/p/1197155653355777.

[57] Kringelbach M.L. (2015). The pleasure of food: Underlying brain mechanisms of eating and other pleasures. Flavour.

[58] Wilson B.M., Stolarz-Fantino S., Fantino E. (2013). Regulating the way to obesity: Unintended consequences of limiting sugary drink sizes. PLoS ONE.

[59] Carl A.E., Taillie L.S., Grummon A.H., Lazard A.J., Higgins I., Sheldon J.M., Hall M.G. (2021). Awareness of and reactions to the health harms of sugary drinks: An online study of U.S. parents. Appetite.

[60] Shomaker L.B., Tanofsky-Kraff M., Savastano D.M., Kozlosky M., Columbo K.M., Wolkoff L.E., Zocca J.M., Brady S.M., Yanovski S.Z., Crocker M.K. (2010). Puberty and observedenergyintake: Boy, cantheyeat!. Am. J. Clin. Nutr..

[61] China Insights Consultancy China Yogurt Industry Bluebook: Low-Temperature Yogurt Segment Crowded with Players, Entering an Era of Product Intensification.

[62] Branum A.M., Rossen L.M., Schoendorf K.C. (2014). Trends in caffeine intake among U.S. children and adolescents. Pediatrics.

[63] Fagan M.J., Di Sebastiano K.M., Qian W., Leatherdale S., Faulkner G. (2020). Coffee and cigarettes: Examining the association between caffeinated beverage consumption and smoking behaviour among youth in the COMPASS study. Prev. Med. Rep..

[64] Berube L., Duffy V.B., Hayes J.E., Hoffman H.J., Rawal S. (2021). Associations between Chronic Cigarette Smoking and Taste Function: Results from the 2013–2014 national health and nutrition examination survey. Physiol. Behav..

[65] Griffith R., O’Connell M., Smith K. (2017). The Importance of Product Reformulation Versus Consumer Choice in Improving Diet Quality. Economica.

[66] Li Y., Teng D., Shi X., Qin G., Qin Y., Quan H., Shi B., Sun H., Ba J., Chen B. (2020). Prevalence of diabetes recorded in mainland China using 2018 diagnostic criteria from the American Diabetes Association: National cross sectional study. BMJ-Br. Med. J..

[67] National Health Commission of China (2020). 2020 China Nutrition and Chronic Diseases Report.

[68] Zhang Z. Opening Address at the Multicenter Investigator Meeting. Proceedings of the 2023 China Obesity Congress.

[69] Rogers P.J., Brunstrom J.M. (2016). Appetite and energy balancing. Physiol. Behav..

[70] WHO (2023). Use of Non-Sugar Sweeteners. WHO Guideline.

